# A second functional furin site in the SARS-CoV-2 spike protein

**DOI:** 10.1080/22221751.2021.2014284

**Published:** 2022-01-04

**Authors:** Yue Zhang, Li Zhang, Jiajing Wu, Yuanling Yu, Shuo Liu, Tao Li, Qianqian Li, Ruxia Ding, Haixin Wang, Jianhui Nie, Zhimin Cui, Yulin Wang, Weijin Huang, Youchun Wang

**Affiliations:** a Division of HIV/AIDS and Sex-transmitted Virus Vaccines, Institute for Biological Product Control, National Institutes for Food and Drug Control (NIFDC), Beijing, People's Republic of China; b National Vaccine & Serum Institute, Beijing, People's Republic of China; c Lead Contact

**Keywords:** SARS-CoV-2, furin, infectivity, S2’ cleavage, cell–cell fusion, pseudovirus

## Abstract

The ubiquitously-expressed proteolytic enzyme furin is closely related to the pathogenesis of SARS-CoV-2 and therefore represents a key target for antiviral therapy. Based on bioinformatic analysis and pseudovirus tests, we discovered a second functional furin site located in the spike protein. Furin still increased the infectivity of mutated SARS-CoV-2 pseudovirus in 293T-ACE2 cells when the canonical polybasic cleavage site (682–686) was deleted. However, K814A mutation eliminated the enhancing effect of furin on virus infection. Furin inhibitor prevented infection by 682–686-deleted SARS-CoV-2 in 293T-ACE2-furin cells, but not the K814A mutant. K814A mutation did not affect the activity of TMPRSS2 and cathepsin L but did impact the cleavage of S2 into S2′ and cell–cell fusion. Additionally, we showed that this functional furin site exists in RaTG13 from bat and PCoV-GD/GX from pangolin. Therefore, we discovered a new functional furin site that is pivotal in promoting SARS-CoV-2 infection.

## Highlights

- Amino acid 814 is the key site for furin in promoting SARS-CoV-2 infection.
- K814A mutation does not affect the activity of TMPRSS2 and cathepsin L.
- K814A mutation affects the cleavage of S2 into S2′ and cell–cell fusion.
- Related coronaviruses RaTG13 and PCoV-GD/GX also possess functional furin site.

## Introduction

Severe acute respiratory syndrome coronavirus 2 (SARS-CoV-2) has caused a global pandemic since 2019 [1]. Its rapid spread and high death rate have had a significant impact on public life throughout the world. However, the mechanisms of virus-host interactions have yet to be elucidated.

As for other coronaviruses, the entry of SARS-CoV-2 to the host cell is mediated by its spike (S) glycoprotein, which plays a decisive role in infectivity [2]. S comprises two functional subunits S1 and S2. The S1 subunit is responsible for the binding of the virus to the host cell receptor, whereas the S2 subunit is involved in membrane fusion of the viral and cellular membranes [3]. For many coronaviruses, S is cleaved at the S1/S2 cleavage site, while the S1 and S2 units remain non-covalently bound in the prefusion conformation [4,5]. After endocytosis of coronavirus by host cells, lysosomal protease further mediates the cleavage of S2 subunit at S2′ cleavage site and releases the hydrophobic fusion peptide to fuse with host cell membrane [5,6].

**Figure 1.**
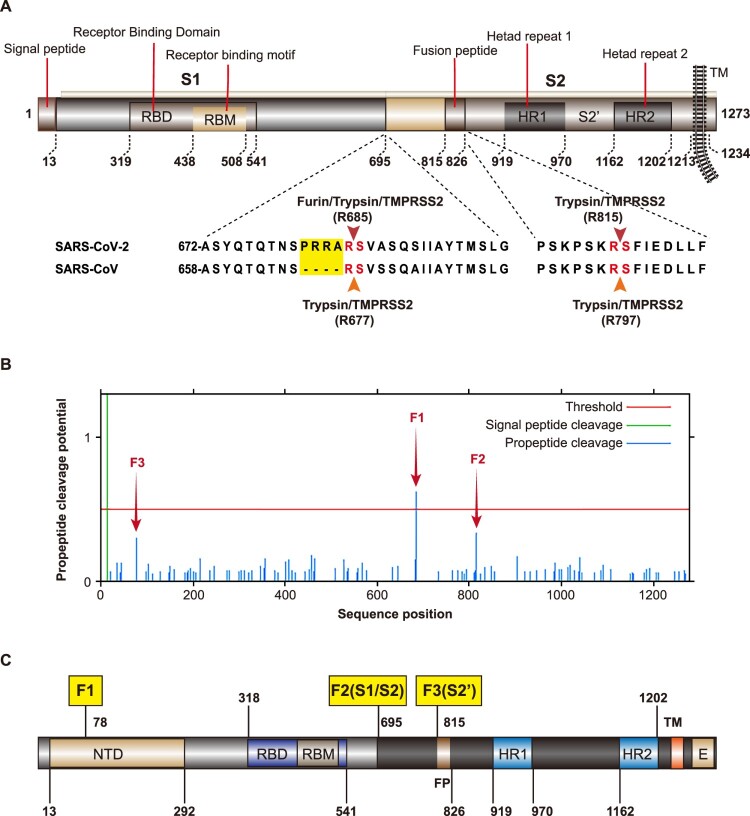
Schematic of furin cleavage sites. A. Schematic of SARS-CoV-2 S protein. The alignment of SARS-CoV and SARS-CoV-2 at S1/S2 and S2′ sites was shown in the lower panel. NTD, N-terminal domain; RBM, receptor-binding motif; FP, fusion peptide; TM, transmembrane domain; CT, C-terminal endodomain. B. Identification of additional furin cleavage sites within the SARS-CoV-2 S protein. ProP 1.0 server (www.cbs.dtu.dk/services/ProP/) was used to carry out the prediction using the furin-specific prediction as the default. C. Schematic of three furin cleavage site predicted by ProP1.0.

The cleavability of glycoproteins is always a prerequisite for viral infectivity and pathogenicity [7]. Host proteases such as furin, transmembrane protease serine 2 (TMPRSS2), cathepsin B (CTSB), and cathepsin L(CTSL) work together for priming and triggering coronavirus S proteins [3]. SARS-CoV was found to utilize TMPRSS2 for entry during “early” pathway, and utilize endosomal CTSL to enter during the “late” pathway [8–10]. Middle East respiratory syndrome (MERS) also utilizes TMPRSS2 and CTSL for spike priming. However, unlike SARS-CoV, MERS also used furin-related proprotein convertases for S priming, as it has an RSVR insert at the S1/S2 boundary [11,12]. In the case of SARS-CoV-2, previous study has shown that SARS-CoV-2 has a unique four-amino acid insertion (681-PRRA-684) in the S protein at nucleotide position 23619-23632. This insertion creates a potential furin cleavage site (682-RRAR-685) for SARS-CoV-2 [13,14] (Figure 1A).

As an ancient proprotein convertase, furin is ubiquitously expressed and regulates normal physiological functioning of cells and processes in virus-infected disease [5]. Furin-mediated cleavage has been reported in numerous evolutionarily diverse virus families, including human immunodeficiency viruses [15], influenza A [16,17], Ebola and Marburg viruses [18,19], Papillomaviruses [20], and Hepatitis B virus [21,22]. Interestingly, most low pathogenic avian influenza A viruses cannot be cleaved by furin as they only have a mono-or dibasic cleavage site, whereas many highly pathogenic avian influenza A viruses (e.g., H5 and H7) can be cleaved by furin due to the insertion of a polybasic cleavage site [23–25]. Furthermore, the glycoprotein of Marburg viruses and human pathogenic Ebolavirus species contains canonical furin cleavage sites (R-X-K/R-R↓) whereas the closely related human asymptomatic Reston virus is processed less efficiently by furin as it carries the suboptimal cleavage site (K-Q-K-R↓) [18,19].

As for SARS-CoV-2, previous studies have reported that S1/S2 cleavage is essential for subsequent S2′ activation via TMPRSS2 for entry in Calu-3 cells but not for S2′ activation via cathepsin L for entry in Vero E6 cells [26,27]. In addition, furin promotes SARS-CoV-2 infection and cell-cell fusion [28]. Loss of furin cleavage attenuates SARS-CoV-2 pathogenesis in both hamsters and hACE2 transgenic mice [29].

During our study of furin in SARS-CoV-2 infection, based on bioinformatic prediction, in addition to the classical furin cleavage site (682-RRAR-685), we found a novel furin cleavage site (K814A) in the S protein of SARS-CoV-2. The role of these sites in SARS-CoV-2 infection, S cleavage, cell–cell fusion, and antigenicity were investigated. Moreover, the corresponding site on other coronaviruses was also tested, including pangolin and bat coronaviruses. Our results provide new clues for the mechanism of furin-mediated cleavage during coronavirus infection.

## Results

### Furin cleavage site prediction

**Figure 2.**
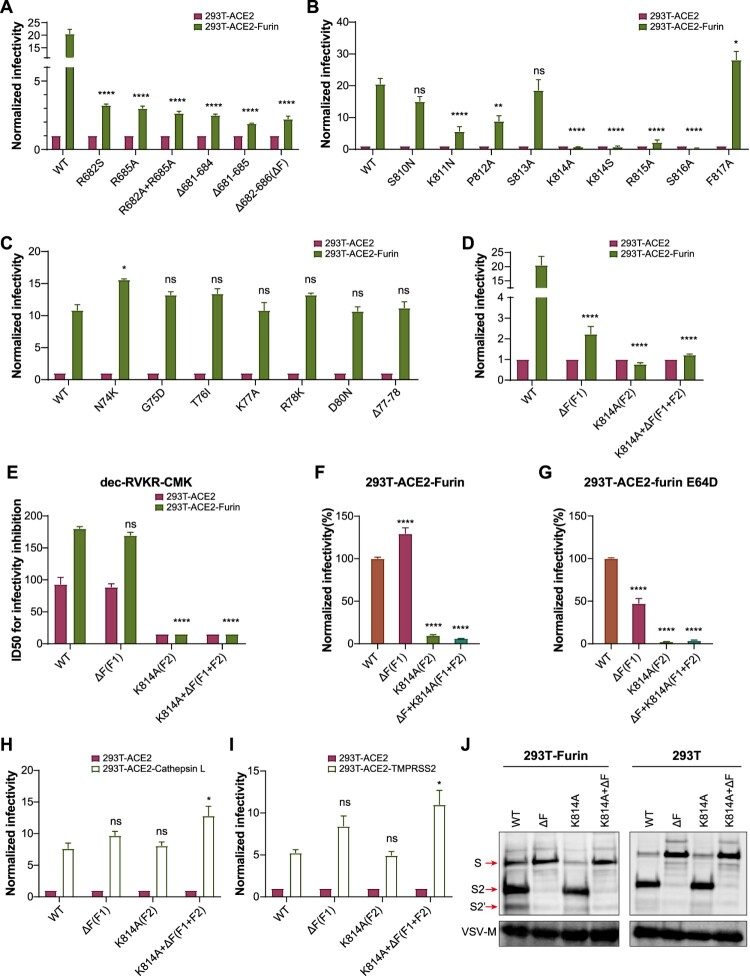
Validation of furin activation sites. A–C. Infectivity analysis of SARS-CoV-2 mutants at F1, F2, and F3 sites. Normalized chemiluminescence signals (in RLUs) in 293T-ACE2-furin cells were calculated by comparing with 293T-ACE2 cells. D. Comparison of F1 and F2 single and double mutations. E. ID50 of furin inhibitor. F–G. Infectivity analysis of F1 and F2 mutations in 293T-ACE2-furin cells, with and without cathepsin inhibitor (E64D). Chemiluminescence signals (in RLUs) were normalized against WT SARS-CoV-2. H–I. Infectivity analysis of F1 and F2 mutants. Normalized chemiluminescence signals (in RLUs) in 293T-ACE2-CathepsinL/TMPRSS2 cells were calculated by comparing with 293T-ACE2 cells. A–I. Data represent the results of three replicate experiments. Values shown indicate means ± SEM. J. WT and mutated SARS-CoV-2 pseudoviruses were centrifuged in sucrose buffer, then resuspended in PBS for SDS-PAGE. Western blotting was performed with mouse anti-S2 polyclonal antibodies. VSV-M was used as an internal control. The statistical tests were comparisons of each pseudotyped mutated virus group with pseudotyped WT virus group.

To study the role of furin in SARS-CoV-2 infection, we used ProP1.0 software (http://www.cbs.dtu.dk/services/ProP/) to analyse the potential furin sites (Figure 1B and 1C). According to the software, SARS-CoV-2 has three possible cleavage sites, among which the highest scoring is F1 (0.62) at NSPRRAR↓S (679–686), followed by F2 (0.333) at PSKPSKR↓S (809–816), and F3 (0.299) at GTNGTKR↓FD (71–79) (Figure 1B and 1C). The F1 site is located at the junction of S1 and S2, and represents the canonical polybasic cleavage site. The F2 site is located at S2′, and the F3 is located at the N terminal domain (NTD) of the S protein (Figure 1B and 1C). Since furin overexpression promotes the infectivity of SARS-CoV-2, to study whether the effect of furin depends on the predicted site, we constructed a series of mutant pseudoviruses around these sites (Figure 2A–C).

### Enhancement of SARS-CoV-2 infectivity by furin overexpression depended on 682–686 and 814 sites

The amino acids near the three predicted sites were mutated sequentially and the infectivity was compared between furin overexpressing or control cells, to examine whether these sites are the key sites of furin. For the canonical polybasic cleavage site F1, we constructed with deletion of several amino acids (Δ682–686, Δ681–684, Δ681–685), and single point (R682S, R685A) mutants. The results showed that although the deletion and mutation of these sites limited the infectivity enhancement caused by furin overexpression to a certain extent, the mutated SARS-CoV-2 still increased the infectivity by approximately two-fold compared with the wildtype SARS-CoV-2 (Figure 2A). Interestingly, when the amino acids at the F2 site (positions 810–817) were mutated one by one, furin no longer enhanced the infectivity of 814–816 mutated SARS-CoV-2 strains (K814A, K814S, R815A, or S816A), whereas K811N and P812A partially affected the furin-enhanced infectivity (Figure 2B). These results suggest that the F2 location is very important for the activity of furin. Finally, we also mutated F3 site. A single K77A point mutant and a combined mutations at position 77–78 were constructed. In addition, natural mutations around this position (N74K, G75D, T76I, R78K, D80N) was also tested. None of these mutations had any effect on furin activity (Figure 2C). Because the mutations of 815A and 816A greatly reduced the infectivity of the virus, we selected the 814A mutant strain to represent the F2 site and ΔF(Δ682–686) to represent the F1 site in the follow-up study. The F3 locus was not studied further due to its insignificant effect. To examine whether there was a synergistic effect between F1 and F2, a double mutant was constructed (F1+F2); however, the results suggested that there was no obvious synergistic effect (Figure 2D). Moreover, furin inhibitor (dec-RVKR-CMK) prevented infection of 293T-ACE2-furin cells by wildtype (WT) SARS-CoV-2 as well as by the ΔF mutated virus but not the K814A mutant (Figure 2E). These results suggest that F2 (K814A) is a novel furin functional sites.

### Effect of Δ682–686 and K814A mutation on Cathepsin L and TMPRSS2

When we compared the infectivity of different mutants in furin-expressing (293T-ACE2-furin) cells, unexpectedly, the infectivity of ΔF was even higher than that of WT (Figure 2F). We speculate that the increased infectivity may be caused by other compensative enzymes. Because TMPRSS2 was not expressed in 293 T cells [3], we first studied the effect of Cathepsin L. After the cells were treated with Cathepsin L inhibitor E64D, the infectivity of ΔF was lower than that of WT (Figure 2H), suggesting that the increase of infectivity in the ΔF mutant may be mediated by Cathepsin L. After the compensatory effect from cathepsin was inhibited, the infectivity of ΔF mutant became lower than that of WT. We further examined whether K814A mutation would influence the function of Cathepsin L. Compared with WT SARS-CoV-2, neither Cathepsin L enhancing SARS-CoV-2 infectivity nor E64D inhibiting SARS-CoV-2 infectivity was shown in the K814A mutant, suggesting that the K814 site may not be the functional site of Cathepsin L (Figure 2H).

As the ΔF and K814A mutations were close to the active sites of TMPRSS2 (685–686 and 815–816), we further determined the effects of these mutations by overexpression of TMPRSS2 in 293T-ACE2 cells. The results indicated that the K814A mutation did not affect the increased infectivity caused by TMPRSS2 overexpression, while the ΔF mutation slightly promoted the enhancement of infectivity caused by TMPRSS2 overexpression (Figure 2I). These results suggest that the K814A mutation affects furin activity only, but not that of other enzymes, whereas the mutation of ΔF not only interfered with the activity of furin, but also promoted the activity of TMPRSS2 and Cathepsin L to a certain extent.

### Effect of Δ682–686 and K814A mutation on furin-mediated S cleavage

We further analysed whether the two sites affected furin-mediated cleavage of SARS-CoV-2 S protein. Western blotting analysis showed that the spike of purified SARS-CoV-2 pseudovirus was no longer cleaved into S1 and S2 when 682-686 site was deleted (Figure 2J), suggesting that the cleavage of S1 and S2 by furin mainly depends on amino acids within the 682–686 site. Furthermore, the proportion of S2 and S2’ in a 293T-furin-overexpressing cell line was higher than that in 293 T cells (line 1 vs line 5). The results suggested that furin over expression could promote the cleavage of both S2 and S2’. Furthermore, in the 293T-furin cell line, the proportion of S2’ in the K814A mutant was lower than that of WT strain (line3 vs line 1), suggesting that K814A mutation affects S2’ cleavage.

## S protein-mediated cell–cell fusion depends on both 682–686 and 814 sites

**Figure 3.**
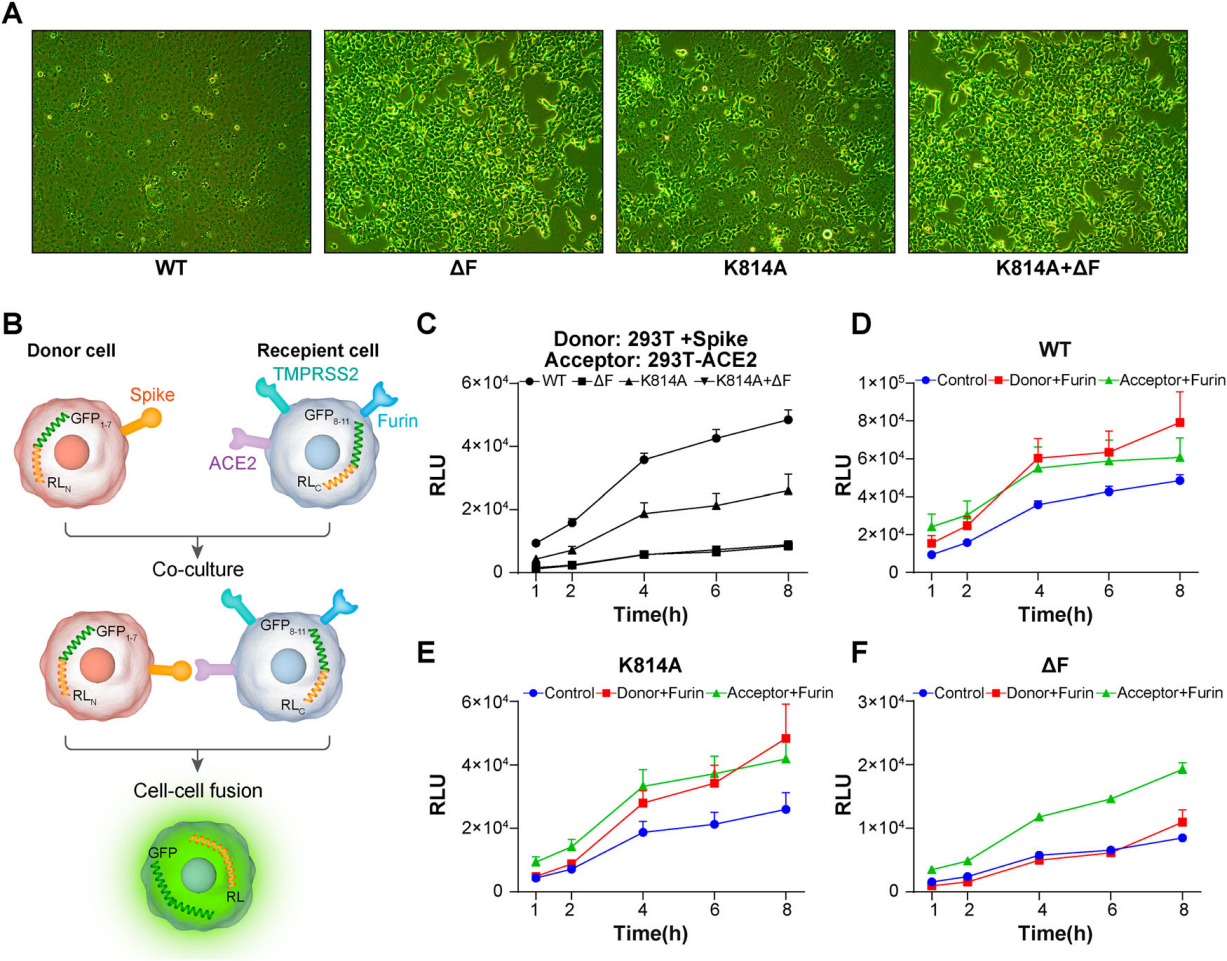
Analysis of furin activation site on cell–cell fusion. A. 293T-ACE2-furin cells were transfected with indicated S-expressing plasmid. The Cell morphology was investigated under bright field microscopy. B. Diagram of dual reporter cell–cell fusion system. 293 T cells were used as donor cells and 293T-ACE2 cells were used as recipient cells. F. Time course curve of cell–cell fusion. RLU signals of Renilla luciferase are shown. Data indicate means ± SEM. Representative results of three independent experiments are shown.

During the study of furin-related site mutation in the S protein, cells were investigated by light microscopy under bright field. Cell–cell fusion was observed when WT SARS-CoV-2 S protein was expressed in 293T-ACE2-furin cells. However, ΔF mutated S protein resulted in very little intercellular fusion (Figure 3A), while the fusion capacity of the K814A mutated S protein was significantly decreased compared with WT S (Figure 3A). Cell-cell fusion was almost completely abrogated when S was double mutated (Figure 3A).

We further established a split *Renilla* luciferase system for cell-cell fusion examination as previously reported [30]. SARS-CoV-2 S protein and ACE2 coupled to a pair of split *Renilla* luciferase proteins were expressed in donor and recipient cells, respectively. Cell-cell fusion was monitored by detecting the activity of the *Renilla* luciferase (Figure 3B). Consistent with the microscopy observations, the Renilla luciferase reporter assay also showed that ΔF significantly reduced cell-cell fusion, while the K814A mutation partially reduced cell-cell fusion (Figure 3C). To further analyse the different roles of furin in donor and recipient cells, we examined the fusion effect of different S mutants by overexpressing the furin protein in donor or recipient 293 T cells, respectively. For WT and K814A mutated S, no differences were observed when furin was expressed either in donor or recipient cells. However, for ΔF mutants, only furin expression in recipient cells promoted cell-cell fusion, suggesting that the furin effect at the F2(814) site only occurs in recipient cells.

### Similar furin active sites on SARS-CoV-2 related coronaviruses RaTG13 and PCoV-GD/GX

**Figure 4.**
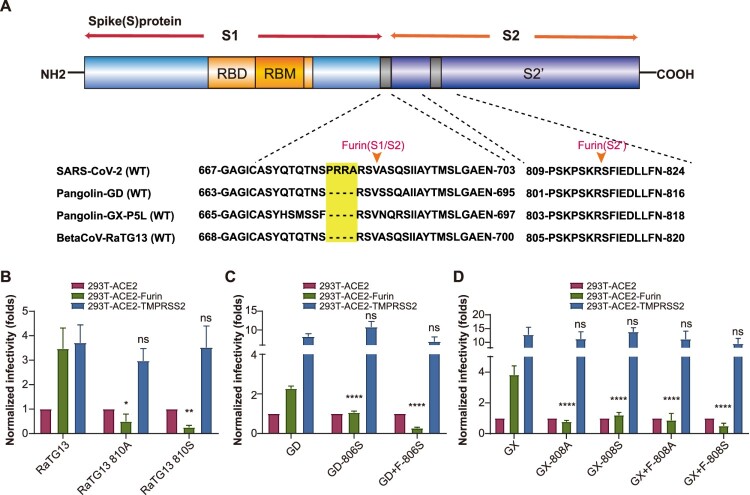
Analysis of furin activation site in other coronaviruses. A. Schematic of SARS-CoV-2 S protein and the alignment of SARS-CoV-2, RaTG13, and PCoV-GD/GX at S1/S2 and S2′ sites. B–D. Infectivity of mutated RaTG13 (B), PCoV-GD (C), and PCoV-GX (D) pseudoviruses in 293T-ACE2, 293T-ACE2-furin, and 293T-ACE2-TMPRSS2 cells. RLU signals were normalized to 293T-ACE2 control cells. +F indicates that PRRA was inserted into the viruses at the S1/S2 site. The statistical tests were comparisons of each pseudotyped mutated virus group with pseudotyped WT virus group.

Coronaviruses RaTG13, identified from bat, and PCoV-GD or PCoV-GX identified in pangolin, are postulated to be closely related to SARS-CoV-2. We further analysed the sequences of these coronavirus. Although none of these viruses contain the canonical polybasic cleavage site at the junction of S1 and S2, all of them include a highly conserved PSKPSKRS sequence in S2, which matches the 809-816 sequence in SARS-CoV-2(Figure 4A). As we expected, the overexpression of furin in 293 T cells enhanced the infectivity of RaTG13, PCoV-GD, and PCoV-GX pseudoviruses (Figure 4B, 4C and 4D). Mutants K810A/S of RaTG13, K806S of PCoV-GD, and K808A/S of PCoV-GX lost the ability to be enhanced by furin, which was similar to the result observed in SARS-CoV-2. We also added the polybasic cleavage site RRAR to the S1/S2 junction of PCoV-GD or PCoV-GX pseudovirus on the K806S or K808A/S mutation background. Furin-mediated infectivity enhancement could not be rescued, which was consistent with our observations in SARS-CoV-2 (Figure 4C and 4D). Furthermore, these mutations did not affect the function of TMPRSS2 (Figure 4B, 4C and 4D). Therefore, these results indicated the presence of a furin activation site in S2′, which was a key site for furin activity, not only in SARS-CoV-2, but also in related coronaviruses from bat and pangolin.

### Effect of Δ682–686 and K814A mutations on antigenicity of SARS-CoV-2

**Figure 5.**
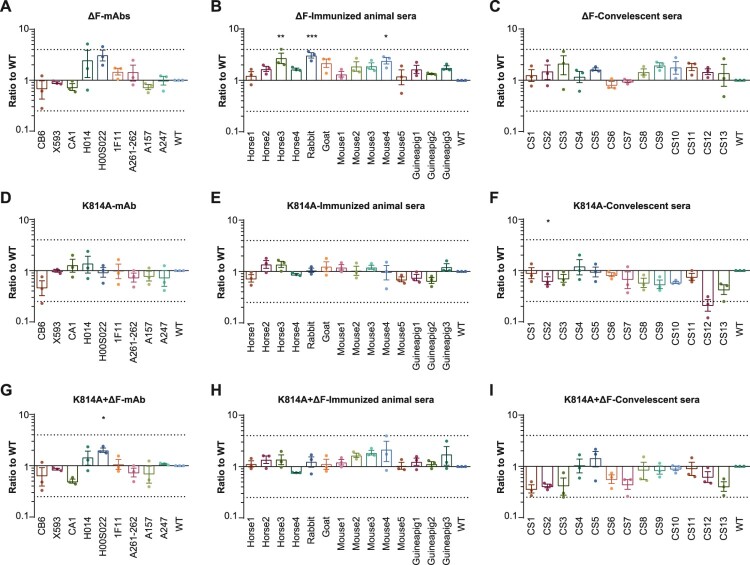
Sensitivity of furin site mutated SARS-CoV-2 to neutralization. ID_50_ ratios normalized against WT are shown as means ± SEM. Dashed lines indicate the threshold of fourfold difference. All experiments were repeated two to four times, depending on sample availability. CS, convalescent sera. The statistical tests were comparisons of ID_50_ of each pseudotyped mutated virus group with ID_50_ of pseudotyped WT virus group.

To explore whether Δ682–686 and K814A affect the antigenicity of SARS-CoV-2, the neutralization characteristics of nine monoclonal antibodies, 12 convalescent sera, and 14 animal immune sera against WT and mutant pseudovirus were tested. Although the ΔF mutation slightly increased neutralization by most of the antibodies and immune sera, the 50% inhibitory dilution (ID_50_) did not increase or decrease more than four-folds compared with the original strain. Therefore, none of the mutants showed significant changes in their neutralization sensitivity (Figure 5).

### Effect of Δ682–686 and K814A mutations on SARS-CoV-2 infectivity of lung cell lines and human ACE2 transgenic mice

**Figure 6.**
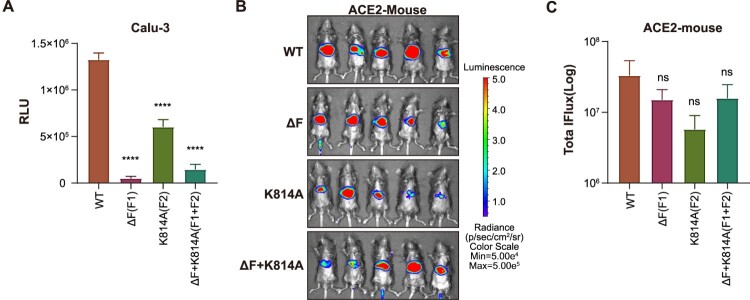
Effect of Δ682–686 and K814A mutations on SARS-CoV-2 infectivity of lung cell line and human ACE2 transgenic mice. A. Infectivity of pseudotyped SARS-CoV-2 mutants in Calu-3 cells. The statistical tests were comparisons of each pseudotyped mutated virus group with the pseudotyped WT virus group. The experiment was repeated twice with three duplicates each time. B. Infectivity of pseudotyped SARS-CoV-2 mutants in human ACE2 transgenic mice. The relative intensities of emitted light are presented as the photon flux values in photon/(sec/cm2/sr) and displayed as pseudocolor images, with colours ranging from blue (lowest intensity) to red (highest intensity). One of two repeated experiments is shown. C. Pseudotyped virus infection in each group as indicated by the total flux values. Statistical significance was assessed by one-way ANOVA with Tukey's post hoc test for multiple comparisons with WT.

The infectivity of the WT or mutant SARS-CoV-2 pseudotyped viruses was also tested in a lung cell line, Calu-3 (Figure 6A). Both the Δ682–686 and K814A mutants showed significantly decreased infectivity in the Calu-3 cells. This result indicates that both 682-686 and K814 sites are important for SARS-CoV-2 infection of lung cells. We then tested the infectivity of pseudotyped WT and mutated SARS-CoV-2 in human ACE2 transgenic C57BL/6 mice. The results showed that the infectivity of the Δ682-686 and K814A mutants both decreased (Figure 6B and 6C). However, the difference is not significant due to the large individual differences in mice.

## Discussion

Since the end of 2019, SARS-CoV-2 has spread extremely quickly around the world. Although great efforts have been made to study vaccines and antiviral drugs, the spread of the virus has not yet been effectively controlled (https://covid19.who.int). The mechanism of SARS-CoV-2 infection is only partially known. S protein hydrolases are the first host proteases affecting virus infection, among which furin protease plays a very important role in SARS-CoV-2 S priming [31]. Furin is associated with the pathogenicity of the virus [32], and SARS-CoV-2 virus titers decreased significantly in furin-deficient cell lines [26]. Furin inhibitor can also block S cleavage, virus replication, and cytotoxicity [33].

The presence of the PRRA insertion at positions 681-684 in the S protein of SARS-CoV-2 has always been a key focus of scientists [13,14]. However, the combination of RRAR is similar to, but slightly different from the classical minimum recognition R-X-K/R-R sequence for furin [34,35]. Currently, there is only one known furin cleavage sequence that matches exactly, which is from the bacterial toxin of *Aeromonas hydrophila* proaerolysin [36,37]. After analysis of more than 130 furin protein substrates, Sun et al. found that the furin cleavage sequence does not rely strictly on the fixed R-X-K/R-R (P4–P1), but rather on a motif of 20 amino acids [38]. P1 refers to the first amino acid residue on the N-terminal end of the cutting site. P2, P4 and P6 are the second, fourth and sixth amino acid residues on the N-terminal end, respectively. P1’ refers to the first amino acid residue on the C-terminal end of the cutting site [38].The study concluded that with the exception of arginine (R) at P1, amino acids at other positions can vary [38]. In particular, P4 does not have to be R but maybe some other aliphatic residue [38]. A positively charged residue at P5 or P6 can also compensate for a lack of positive charged at P4 to a certain extent [38]. In addition, K/R at P2 position is not necessary, because more than 20% of the furin sensitive sequences are not K/R at this position [38]. Some small amino acid residues such as glycine or alanine can also be selected at P2 [38]. Although the P3 position is considered to be inclined to positively charged amino acids, it is not in the binding pocket for furin [38]. Theoretically, any amino acids could be used at P3. Therefore, both the RRAR (P4–P1) of 682-685 and the KPSKR(P5–P1) of 811–815 meet the active or catalytic requirements of furin to a certain extent. Consistent with previous results [28,39], our study shows that after the deletion of 682–686, S protein was no longer cleaved into S1 and S2. Furthermore, we found that furin also act on the site around K814 in the S2′ domain and promoted the cleavage of S2 into S2’. Essalmani et al. also suggested that furin can cleave KPSKR↓S (811–815) *in vitro*, although the proteolytic efficiency was much lower than that of RRAR↓SV (682–686) [39]. Interestingly, although the K814 site seems to be responsible for only a two-fold increase of SARS-CoV-2 infectivity by furin (the infectivity of the Δ682–686 mutant can still increase twofold after furin overexpression) and is only related to the S2′ cleavage. The K814A single mutation led to a complete loss of the furin-mediated infectivity enhancement, suggesting its important role during furin functioning (Figure 2D).

In addition, we also compared the function of furin in RaTG13 and PCoV-GD/GX, MERS-CoV, and SARS-CoV. Our results indicate that furin may function on the S2’ region of RaTG13 and PCoV-GD/GX and promote the infectivity of these coronaviruses. As the most lethal coronavirus, MERS-CoV was suggested to have two furin activation sites in its S protein [12,40]. The site between S1 and S2 is RSVR, and its S2’ region sequence is RSAR [12]. It was discovered that MERS-CoV is first catalysed into S1 and S2 by furin during the process of virus formation [12]. S2 is further cleaved into S2’ by furin after the virus enters the cells [12]. Our results show that the SARS-CoV-2 pseudovirus was partially cleaved into S1, S2, and S2’ during virus assembly, which was similar to MERS-CoV. Furthermore, overexpression of furin increased S2’ cleavage. Cell-cell fusion studies suggested that both the K814A site and the 682-686 sites are important for furin-enhanced cell-cell fusion, with the 682-686 sites necessary for furin functioning in donor cells.

The cleavage site of SARS-CoV S protein is SLLR for S1 and S2, and KPTKR for S2’ [41]. It is thought that furin does not affect SARS-CoV [42]. Belouzard et al. found that when the canonical polybasic furin cleavage sequence was artificially introduced into S2’, furin could effectively cleave the mutated S and significantly increase the activity of cell–cell fusion [8]. Our results indicate that overexpression of furin can also increase SARS-CoV pseudotyped virus infection in 293T-ACE2 cells, although not as much as in SARS-CoV-2 (about fivefold, Figure S1A). When the K814 corresponding site of SARS-CoV (K809) was mutated (K to A), furin overexpression cannot increase the infectivity to 293T-ACE2 cells anymore. However, no significant difference was found between the WT and K809A mutated SARS-CoV when the lung cell line Calu-3 and the human ACE2 transgenic mice were tested (Figure S1B–D).

Furin-deficient cells constructed by the CRISPR technique can still become infected by SARS-CoV-2 virus, suggesting that proteases other than furin also play an important role in SARS-CoV-2 infection [28]. A previous study suggested that CTSL can also promote SARS-CoV-2 infection [13]. Interestingly, the infectivity of the Δ682-686 mutant in 293T-ACE2 cells was stronger than that of WT. This enhancement disappeared after the addition of E64D inhibitor, suggesting that ΔF may enhance the enzyme activity of CTSL. As the exact cutting position of CTSL is still unknown, whether the mutation of Δ682-686 affects the catalytic activity of CTSL directly, or whether it is due to increased S protein entering the endosome pathway warrants further research. In addition, E64D is not only the inhibitor of CTSL but also inhibits other cathepsins. Thus, other cathepsins may also play a role during SARS-CoV-2 infection. Although TMPRSS2 is expressed only in epithelial cells such as the respiratory tract, it is also considered to be the key protease for S protein catalysis of SARS-CoV, MERS-CoV, and SARS-CoV-2 [3]. We, therefore, studied the effect of furin site mutation on TMPRSS2. The results suggested that although the introduced mutations are near the theoretical cleavage site of TMPRSS2, they have little effect on the promotion of virus infection by TMPRSS2 in 293T-ACE2 cells.

The emergence of SARS-CoV-2 variants leads to changes in virus antigenicity and infectivity, which has become a new challenge for virus prevention and control [43,44]. Both Alpha and Delta variants have mutations near the classical furin cleavage site (P681H/R) [45]. Interestingly, the transmissibility of Alpha and Delta variant is significantly increased [45–47]. Previous studies suggested that the P681H mutation enhanced the cleavage of S protein by furin, while P681R promoted cell-cell fusion [48,49]. These changes may be related to the increased transmission of the virus. Our results suggest that K814 is also an important site for furin activation, which indicated that attention should be paid to the natural mutation of K814 and those near the K814 site. Since K814 mutation has not widely spread in nature yet, whether the mutation causes decreased infectivity, and whether it might lead to mild or asymptomatic diseases need more real-world data to support.

All results in this study were based on pseudotyped SARS-CoV-2 viruses, which have been widely used to study the infectivity and antigenicity of SARS-CoV-2, as well as the cleavage of the spike protein [3,43]. Although pseudotyped virus-based assays offer great advantages over the wild type virus-based methods, as they are much safer to handle and easier to be mutated or modified, authentic virus strains and primary human respiratory epithelial cells would be better to mimic the real situation in humans.

In conclusion, this study proved that the K814 site of the S protein may be another functional site of furin-mediated cleavage. Although the presence of PRRA at position 682–685 determines whether S protein is cleaved into S1 and S2, whether furin can promote SARS-CoV-2 infection appears to be related to both 682–685 and K814 in S2′. These results provide insight into a new mechanism of furin-mediated SARS-CoV-2 S priming and reveal a potential target for antiviral therapy.

## Materials and methods

### Plasmids

The S protein expression gene of SARS-CoV-2 (Wuhan-Hu-1, GenBank: MN908947) PCoV-GD (GISAID: EPI_ISL_410721), PCoV-GX (GISAID: EPI_ISL_410540), and RaTG13 (GISAID: EPI_ISL_402131) were cloned into the pcDNA3.1 plasmid to construct pcDNA3.1-SARS-CoV-2-S, pcDNA3.1-GD, pcDNA3.1-GX and pcDNA3.1-RaTG13, respectively. Site-directed mutagenesis of S was performed as we described previously [50]. FLAG-tagged ACE2 protein (GenBank: NP_001358344.1) was cloned to pLV (*E. coli*, VB200421-1213bkd; Vector Builder, China). Myc-tagged furin protein (GenBank: NP_002560.1) was cloned to pLV (*E. coli*, VB200420-1476wpj; Vector Builder). HA-tagged TMPRSS2 protein (GenBank: NP_005647.3) was cloned to pLV (*E. coli*, VB200421-1130ffw; Vector Builder). EGFP-tagged CTSL protein (GenBank: NP_001187996.1) was cloned to pLV (*E. coli*, VB200727-1121jwn, Vector Builder), and the dual split cell–cell fusion system (GFP_1–7_ RL_N_/GFP_8–11_ RL_C_) was constructed as described by Kondo et al. [30]. The primers used for mutagenesis are listed in Table S1.

### Cells

293 T (CRL-3216; American Type Culture Collection [ATCC], Manassass, VA), Calu-3 (HTB-55; ATCC), and Huh-7 (Cat0403; Japanese Collection of Research Bioresources) cells were used in this study. 293T-hACE2, 293T-ACE2-furin, 293T-ACE2-TMPRSS2, and 293T-ACE2-CTSL stably expressing cells were constructed by co-transfection of the pLV plasmids with pCMV-Δ8.91 and pMD2.G in 293 T cells and selected with blasticidin (15 µg/mL), hygromycin B (150 µg/mL), puromycin (2 µg/mL) or puromycin (2 µg/mL) respectively. Expression of ACE2, furin, TMPRSS2, or CTSL was determined using quantitative (Q) PCR and Western blotting (Figure S2). Cells were cultured with Dulbecco's modified Eagle medium (DMEM, high glucose; HyClone, Logan, UT). One hundred units per milliliter of penicillin-streptomycin solution (Gibco, Germany), 20 mM N-2-hydroxyethylpiperazine-N-2-ethane sulfonic acid (HEPES, Gibco), and 10% fetal bovine serum (FBS, Pansera ES; PAN-Biotech, Aidenbach, Germany) were added to the culture medium. Lipofectamine 2000 (Invitrogen, Waltham, MA) was used as transfection reagent. Primers used for QPCR identification are listed in Table S1.

### Antibodies and reagents

Anti-HA-Tag antibody (sc-7392; Santa Cruz, Dallas, TX) was used to detect TMPRSS2 and anti-c-Myc antibody (sc-40, Santa Cruz) was used to detect furin, while anti-ACE2 (10108-T60; Sino Biological Inc., Beijing, China) and anti-CTSL (10486-RP02 Sino Biological Inc.) antibodies were used to detect ACE2 and CTSL, respectively. Anti-S2 antibody against SARS-CoV-2 S protein was generated in-house by immunizing mice with recombinant S2 protein. The anti-VSV M protein antibody was purchased from KeraFast (EB0011; Boston, MA). Horseradish peroxidase (HRP)-conjugated goat anti-mouse IgG (CW0102S; CWbiotech, Beijing, China) and HRP-conjugated goat anti-rabbit IgG (SSA004; Sino Biological Inc.) were used as secondary antibodies. Nine monoclonal antibodies against SARS-CoV-2 S protein were used in the neutralization assays, of which CB6 and CA1 were gifts from Dr. Jinghua Yan of University of Chinese Academy of Sciences [51]; X593 was from Dr. X. Sunney Xie of Peking University [52]; H014 and H00S002 were from Dr. Liangzhi Xie of Sino Biological Company [53]; 1F11, A261–262, A157, and A247 were from Dr. Linqi Zhang of Tsinghua University [54]. Furin inhibitor (Decanoyl-RVKR-CMK) was from R&D Systems (Bristol, UK, Cat#: 3501) Cathepsin inhibitor (E64D) was from Apexbio (Shanghai, China, Cat#: A1903).

### Convalescent sera

Convalescent serum samples from SARS-CoV-2 (Wuhan-Hu-1) infected patients were provided by Dr. Xiaowang Qu from Nanhua University. Written informed consent was obtained from all patients before blood collection. The study protocol involving convalescent serum samples complied with the Declaration of Helsinki principles for ethical research.

### Sera from immunized animals

Animals were handled under institutional (National Institutes for Food and Drug Control [NIFDC], Beijing, China) guidelines for laboratory animal care and use. The immunization protocol was described in our previous paper [55,56] and was approved by Animal Care and Use Committee at the NIFDC.

### Pseudoviruses

Pseudotyped viruses of SARS-CoV-2 mutants were constructed by the methods described in our previous study [57]. Briefly, pcDNA3.1.VSV G plasmid was transfected into 293 T cells using lipofectamine 3000 (Invitrogen). Meanwhile, the G*ΔG-VSV (VSV G pseudotyped virus, EH1020-PM, Kerafast) was added to the cell culture supernatant. The cell culture medium was changed 6–8 h later. The culture supernatant was harvested 24 h and 48 h later for pseudotyped virus, which were then filtered, aliquoted, and stored at –80°C.

### Infectivity assay

WT or mutant SARS-CoV-2 pseudoviruses were diluted and mixed with 293T-ACE2 or indicated cells and incubated at 37°C with 5% CO_2_ for 24 h. The britelite plus reporter gene assay system (PerkinElmer, Waltham, MA) and PerkinElmer Ensight device were used to examine chemiluminescence signals which expressed as relative luminescence units (RLUs). Each experiment was repeated three times in duplicate wells.

### Proteolytic cleavage analysis

WT or mutant SARS-CoV-2 pseudoviruses were purified by 25% sucrose density gradient centrifugation at 100,000×*g* for 3 hours. The pellet was then re-suspended in PBS and mixed with loading buffer for sodium dodecyl sulfate-polyacrylamide gel electrophoresis (SDS-PAGE) and Western blotting analysis.

### Cell–cell fusion assay

293 T cells transfected with S and GFP_1–7_ RL_N_ plasmids were used as donor cells. 293T-ACE2 cells were transfected with the GFP_8–11_ RL_C_ plasmid as receptor cells. The cells were detached with trypsin 24 h after transfection, mixed at a 1:1 ratio and seeded into 96-well plates. EnduRen live cell substrate (E6481; Promega, Madison, WI) and an Ensight device (PerkinElmer) were used for luciferase activity detection.

### Neutralization assay

Monoclonal antibodies or serum samples were pre-diluted to certain initial concentrations and serially diluted. Samples were then mixed with pseudotyped virus, and pre-incubated at 37°C for 1 hour. Huh-7 cells were added to each well of the 96-well plate and incubated at 37°C with 5% CO_2_ for 24 hours. RLU signal was detected as for the infectivity assay described above. The ID_50_ was calculated using the Reed–Muench method.

### Animal experiments

The infectivity of pseudotyped SARS-CoV-2 and SARS-CoV to mouse were performed (WT and furin site mutants) in human ACE2 transgenic C57BL/6 mice by bioluminescent imaging (BLI) assay. The transgenic mice were generated by using CRISPR/Cas9 knockin technology as previous reported [58]. The 4-5-week-old mice (weight 13–17 g) were injected with 1.5 × 10^6^ TCID_50_ pseudotyped virus per mouse via tail vein injection. Four to five mice were used in each group. Bioluminescence was measured 1-day post-infection and visualized in pseudocolor. The BLI analysis was performed with the IVIS Lumina Series III Imaging System (PerkinElmer, Baltimore, MD). The Living Image software (Caliper Life Sciences, Baltimore, MD) was used to analyse the regions of interest. The data are presented as the total fluxes in photons/s.

### Statistical analysis

GraphPad Prism 8 (GraphPad, San Diego, CA) was used for statistical analysis. A *t-*test was used for comparison between two experimental groups; one-way ANOVA and Holm–Sidak multiple comparisons tests were used for comparison of several groups. Values are shown as means ± SEMs. *P < 0.05, **P < 0.01, ***P < 0.005, and ****P < 0.001.

## Supplementary Material

Supplemental Material

Supplemental Material

Supplemental Material
