# Supplementary figures and images for: A second functional furin site in the SARS-CoV-2 spike protein
Source: Emerg Microbes Infect. 2022 Jan 4;11(1):182–94. doi: 10.1080/22221751.2021.2014284 (PMC8741242; doi:10.1080/22221751.2021.2014284)

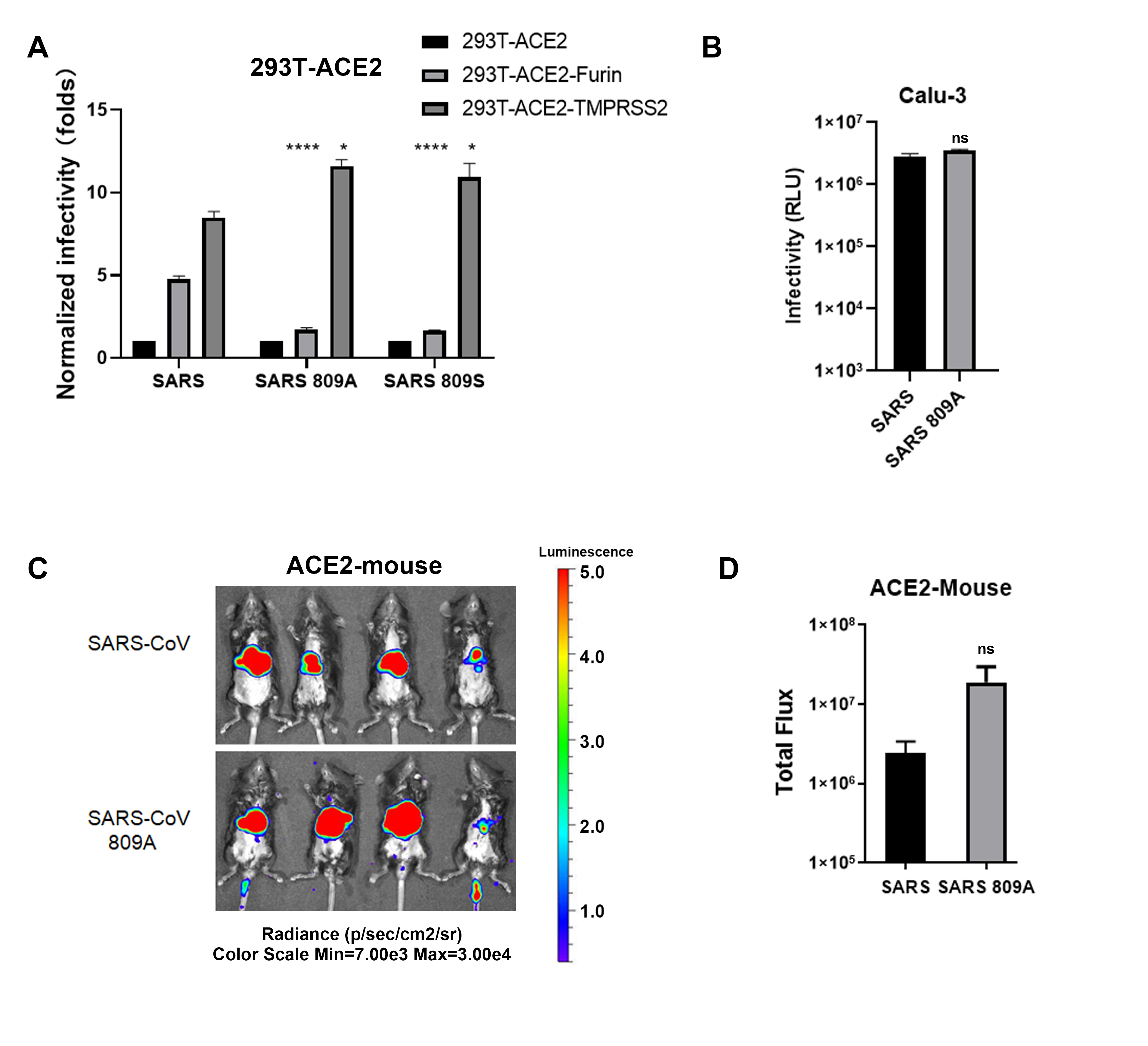

Supplement: Supplemental Material [file TEMI_A_2014284_SM5077.tif]

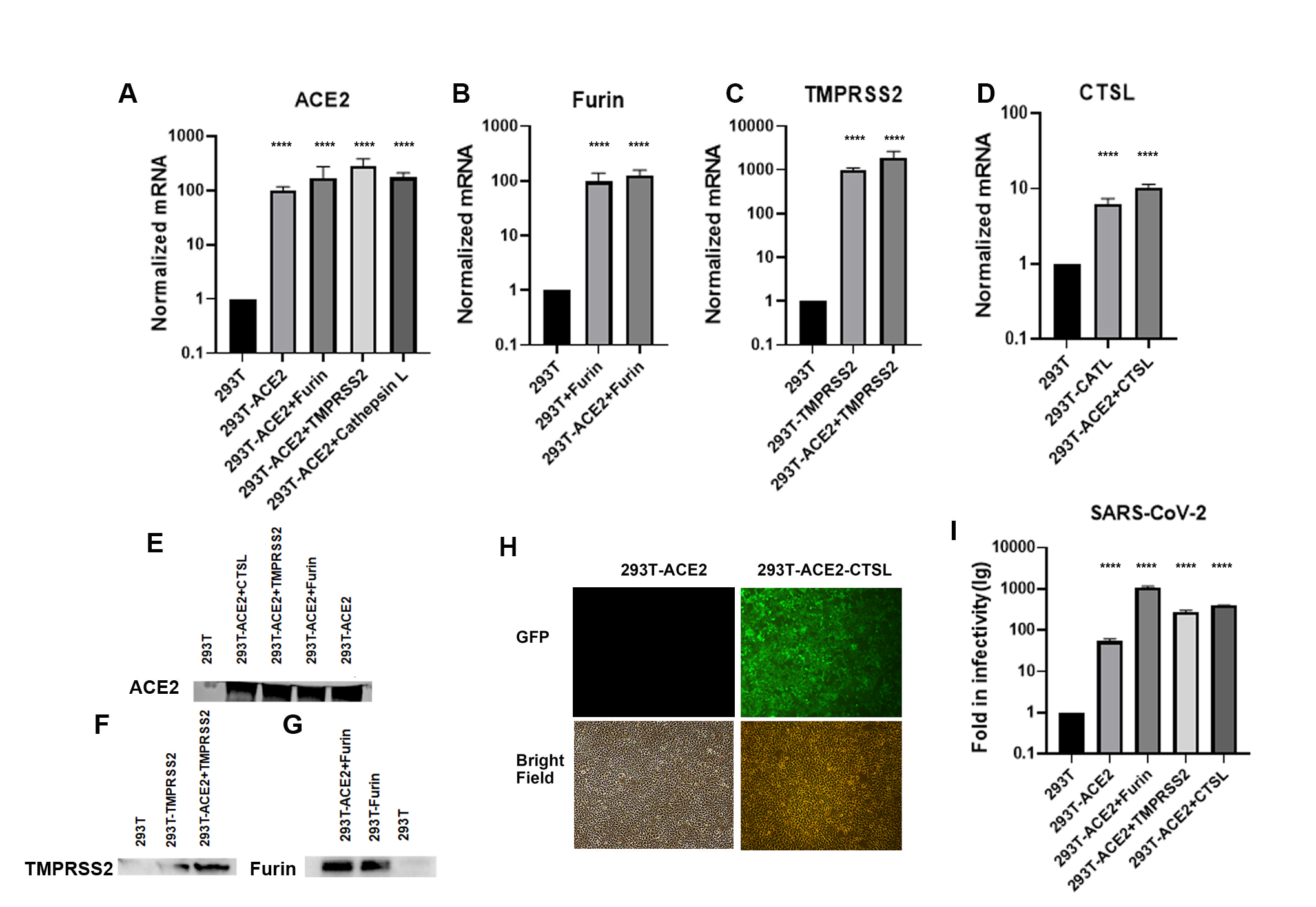

Supplement: Supplemental Material [file TEMI_A_2014284_SM5052.tif]
